# Evaluating the effect of magnesium supplementation and cardiac arrhythmias after acute coronary syndrome: a systematic review and meta-analysis

**DOI:** 10.1186/s12872-018-0857-6

**Published:** 2018-06-28

**Authors:** Shirvan Salaminia, Fatemeh Sayehmiri, Parvin Angha, Koroush Sayehmiri, Morteza Motedayen

**Affiliations:** 1 Department of Cardiac Surgery, Yasuj University of Medical Science, Yasuj, Iran; 2 Proteomics Research Center, Shahid Beheshti University of Medical Sciences, Tehran, Iran; 3 Social Determinants of Health Research Center, Yasuj University of Medical Sciences, Yasuj, Iran; 4 Department of Social Medicine, School of Medicine, Ilam University of Medical Sciences, Ilam, Iran; 5 Department of Cardiology, Faculty of Medicine, Zanjan University of Medical Sciences, Zanjan, Iran

**Keywords:** Serum magnesium level, Arrhythmias, Atrial, Ventricular, Meta-analysis

## Abstract

**Background:**

Atrial and ventricular cardiac arrhythmias are one of the most common early complications after cardiac surgery and these serve as a major cause of mortality and morbidity after cardiac revascularization. We want to evaluate the effect of magnesium sulfate administration on the incidence of cardiac arrhythmias after cardiac revascularization by doing this systematic review and meta-analysis.

**Methods:**

The search performed in several databases (SID, Magiran, IranDoc, IranMedex, MedLib, PubMed, EmBase, Web of Science, Scopus, the Cochrane Library and Google Scholar) for published Randomized controlled trials before December 2017 that have reported the association between Magnesium consumption and the incidence of cardiac arrhythmias. This relationship measured using odds ratios (ORs) with a confidence interval of 95% (CIs). Funnel plots and Egger test used to examine publication bias. STATA (version 11.1) used for all analyses.

**Results:**

Twenty-two studies selected as eligible for this research and included in the final analysis. The total rate of ventricular arrhythmia was lower in the group receiving magnesium sulfate than placebo (11.88% versus 24.24%). The same trend obtained for the total incidence of supraventricular arrhythmia (10.36% in the magnesium versus 23.91% in the placebo group). In general the present meta-analysis showed that magnesium could decrease ventricular and supraventricular arrhythmias compared with placebo (OR = 0.32, 95% CI 0.16–0.49; *p* < 0.001 and OR = 0.42, 95% CI 0.22–0.65; p < 0.001, respectively). Subgroup analysis showed that the effect of magnesium on the incidence of cardiac arrhythmias was not affected by clinical settings and dosage of magnesium. Meta-regression analysis also showed that there was no significant association between the reduction of ventricular arrhythmias and sample size.

**Conclusion:**

The results of this meta-analysis study suggest that magnesium sulfate can be used safely and effectively and is a cost-effective way in the prevention of many of ventricular and supraventricular arrhythmias.

## Background

Coronary artery disease is one of the major causes of death in most industrialized and also other countries. Despite newer medical treatments as well as interventional and surgical techniques, mortality is still significant. In addition to medical treatments for this disease, many patients with the coronary artery disease need surgical treatment. Coronary artery bypass graft is an effective procedure to reduce or eliminate symptoms of angina. However, despite being effective, it also has special complications during and following surgery. One of the most common early complications after open heart surgery is atrial and ventricular arrhythmias, which leads to increased mortality and morbidity in the postoperative period. This complication by increasing hospital stay, it also raises the involved economic costs [1]. The use of cardiopulmonary bypass, as one of the essentials for coronary bypass graft, results in decreased serum magnesium level. Hypomagnesemia is a relatively common electrolyte disorder in hospitalized patients[2] with associated arrhythmias. The arrhythmias which caused by magnesium deficiency are resistant to both antiarrhythmic drugs and cardioversion[3]. As a result, the addition of magnesium sulfate to compensate for Hypomagnesemia could be a method for preventing arrhythmias. During cardiopulmonary bypass, total Magnesium concentration reduces due to the ultrafiltration and also hydration with Albumin and other blood products. However, increasing renal excretion of magnesium does not occur during bypass[4].

Magnesium deficiency presents among about 71% of patients who underwent Cardiopulmonary bypass (CPB) [5]. In many studies intraoperative addition of the magnesium sulfate was beneficial. These benefits were decreasing postoperative arrhythmia rate; lowering the rate of postoperative hypertension and reducing postoperative electrocardiographic changes [6]; increasing coronary blood flow and increased cardiac indexes [7], reduced inflammatory response [8], decreased platelet function [9], and reduced mortality [10].

There is evidence to suggest that low magnesium level could relate to the incidence of ventricular arrhythmias after cardiac surgery and may reduce postoperative ventricular arrhythmia. However, there are different opinions about the relationship between magnesium level and the Arrhythmia in patients with acute coronary syndromes [11]. Many studies performed in this regard and some of them showed relation.

In this systematic review and meta-analysis study, it has been tried to integrate the results of studies that investigate the effect of magnesium sulfate on cardiac arrhythmias. The fundamental aim is to provide a safe and effective way for prevention of cardiac arrhythmias and its complications.

## Methods

### Data sources and search strategy

The present study is a meta-analysis of all data resources about the effect of magnesium sulfate on cardiovascular events after coronary revascularization. The study conducted by the review and meta-analysis of existing electronic sources between 1986 and 2017, including SID, Magiran, IranDoc, IranMedex, MedLib, PubMed, ISI, Web of Science, Scopus, and Google Scholar. The selected studies must evaluate the effect of magnesium sulfate on cardiac arrhythmias and mortality after cardiac revascularization and in both Farsi and English languages. Our keywords were magnesium sulfate, bypass, coronary artery, arrhythmia, atrial, ventricular and their Persian equivalents and with all their possible combinations. Also, all titles and references of the selected articles used as additional search tools for relevant studies.

### Study selection) inclusion and exclusion criteria)

This review considered all randomized controlled trials which evaluated the relationship between magnesium sulfate with cardiac arrhythmias. Inclusion criteria were as follow: any study carried out in the patients with acute coronary syndromes (all patients undergoing CABG or PCI and also medically treated subjects); evaluated the correlation of the arrhythmias and serum magnesium sulfate; compared the administration of magnesium to a placebo group, and reported clinical events such as incidence and type of arrhythmias (supraventricular arrhythmias / ventricular arrhythmias) and or mortality. Any study excluded if was in conjunction with another heart disease; not reported the incidence of arrhythmias, were case studies, and those not compared magnesium with placebo. Also, studies excluded if published in languages other than English or Persian, those that were meta-analyses or systematic reviews, and those that presented insufficient data or were duplicate publications.

### Data extraction

Data extracted after study appraisal. Quality assessment was assayed by NOS scale (New Ottawa Scale). For this purpose, a form designed with multiple pieces of information and the fundamental data needed for analysis (participants, interventions, outcomes, and study quality). The following information extracted and recorded: the first author, the year of publication, study location, number of patients in the treatment and control groups, average group age, group gender, the systemic magnesium dose, mortality, the incidence and types of arrhythmias (supraventricular and ventricular arrhythmias) and mortality. Two authors evaluated independently all included trials and extracted data on the basis of a standard protocol extraction. In cases which needed more information, the articles’ writers contacted for supplementary data or further elucidation. Disagreements about study eligibility resolved by group discussion. The data entered into Microsoft Excel.

### Statistical analysis

One of the main objectives of this study was to evaluate the incidence of arrhythmia; therefore, the binomial distribution used to calculate the variance in each study and Weighted Average used to combine arrhythmia rate in different studies. Each study weight was proportional to its inverse of the variance. The odds ratio (OR) with a confidence interval of 95% computed as the effect measure for both individual trials and pooled estimates. For dichotomous data (adverse effects), the Peto odds ratio (to account for the potential of 0 counts in the cells for low-frequency outcomes) and 95% CI reported.

Statistical heterogeneity evaluated in studies using Q and I^2^ Cochran statistics. When the results of studies were heterogeneous, the analysis performed using a random-effects model. Also, wherever there was no heterogeneity for the outcome, the fix effects model used to pool analysis and verses. Thus, in this meta-analysis, two main approaches used: the fix effects model and the random-effects model.

Subgroup analyses carried out to investigate the dosage effect of magnesium used (< 10 g, > 10 g) and clinical settings (surgery and not surgery) on the evaluated outcomes. We conducted a meta-regression analysis with sample size, mean age and magnesium dose as independent variables and log OR as the dependent variable to assess sources of heterogeneity. Integrated estimations and the related confidence interval of 95% evaluated using forest plots as visuals. Funnel plots and Egger test used to examine publication bias. Values of *p* < 0/05 considered as valid for heterogeneity tests. The analysis conducted with software R (version 3.2.1) and STATA (version 11.1). All statistical tests were two-sided.

## Results

### Selected articles

**Fig. 1:**
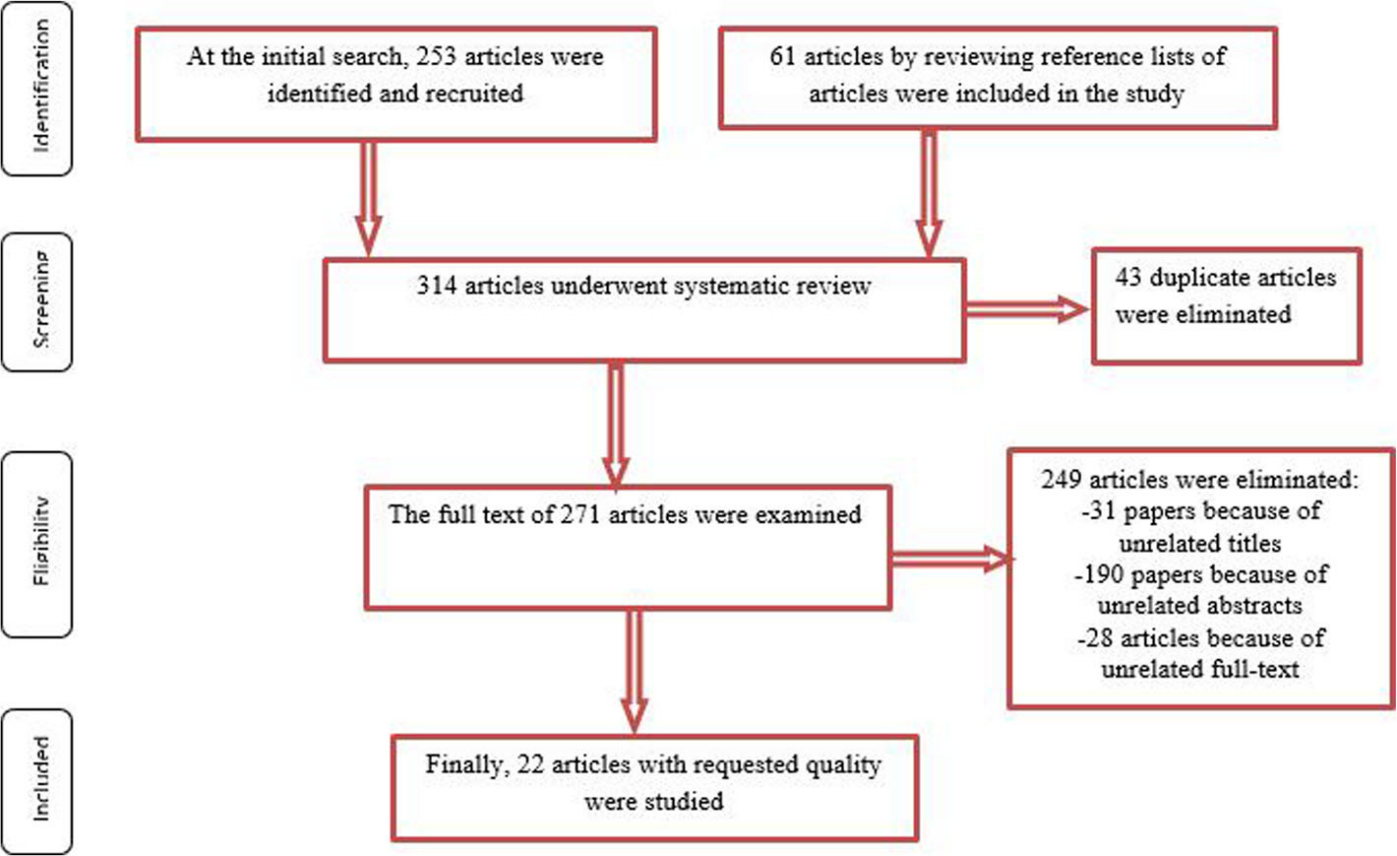
Flowchart steps of the systematic review and meta-analysis

In this meta-analysis, we first identified 253 clinical trials. By manual search of the bibliographies and reference lists of these articles, we identified another 61 additional articles. Altogether, 314 articles identified through the literature search and 43 of them eliminated because of being repetitive. Article selection completed considering three steps: title, abstract and the full text. After the initial screening of clinical trials, 31 papers excluded with unrelated titles. In a secondary screening of the abstracts, 190 papers excluded with unrelated abstracts. In the next step and after a full-text review, another 28 article excluded; finally, twenty-two published articles from 1986 to 2017 [12–33] selected to be appropriate for the final analysis (Fig. 1). Collected number of participants were 6061 individuals, which contained 2987 in Magnesium and 3074 in the placebo group respectively. Table 1 summarizes the characteristics of the eligible studies.

**Table 1:** General characters of studies entered meta-analysis

Authors	Year of Publication	Country	Clinical settings	number		Mean Age		Treatment
				Mg	P	Mg	p	
L.F. Smith^12^	1986	U.K.	Not surgery	92	93	59.7 ± 0.9	58.4 ± 1.1	65 mmol MgSO4 over 24 h
Rasmussen HS^13^	1987	Denmark	Not surgery	55	75	64.6	67.6	12 mmol MgSO4 over 24 h
Shechter.M^14^	1990	Israel	Not surgery	50	53	64 ± 10	63 ± 11	22 g MgSO4 over 24 h
M.Thiigersen.A^15^	1993	Sweden	Not surgery	54	55	67 ± 10	67 ± 11	50 mmol MgSO4 over 20 h
Roffe.C^16^	1994	U.K.	Not surgery	22	26	65.7 ± 12.7	60.2 ± 9.7	73 mmol MgSO4 over 24 h
Bhargava.B^17^	1995	India	Not surgery	40	38	58 ± 10	56 ± 8	65 mmol MgSO4 over 24 h
Karmy-Jones.R^18^	1995	Canada	CABG	46	54	64.5 ± 7.9	60.2 ± 11.9	2.4 g MgSO4 over 24 h
Shakerinia.T^19^	1996	Iran	CABG	25	25	67.2 ± 8.3	64.9 ± 6.7	15 mmol/L MgSO4 over 24 h
Raghu.C^20^	1999	India	Not surgery	169	181	52.9 ± 11	53.1 ± 10.8	18 g MgSO4 over 24 h
Parikka.H^21^	1999	Finland	Not surgery	31	26	60 ± 6	59 ± 6	70 mmol MgSO4 over 24 h
Treggiari-Venzi MM^22^	2000	Switzerland	CABG	47	51	65	65	4 g MgSO4 over 24 h
M. Santoro.G^23^	2000	Italy	Angioplasty	75	75	60 ± 11	60 ± 12	7 g MgSO4 over 5 h
Toraman F^24^	2001	Turkey	CABG	100	100	62 ± 6.7	61.4 ± 8.7	0.8 g MgSO4 over 24 h
Nakashima H^25^	2004	Japan	Angioplasty	89	91	67 ± 11	69 ± 11	20 g MgSO4 over 24 h
Ebadi.A^26^	2008	Iran	CABG	81	81	61.6 ± 5.5	61.7 ± 8.5	2 g MgSO4 over 24 h
Tiryakioglu.O^27^	2009	Turkey	CABG	64	64	58 ± 8	57.6 ± 8.8	3 g MgSO4 over 24 h
Cook RC^28^	2009	Canada	CABG	462	465	–	–	5 g MgSO4 over 24 h
MoeenVaziri MT^29^	2009	Iran	CABG	25	26	60.1 ± 8.9	60.8 ± 10.5	30 mg/kg MgSO4 in 5 min
Tabari.M^30^	2009	Iran	CABG	60	60	61.3 ± 0.6	58.4 ± 10.3	4.5 g MgSO4 over 24 h
Mhaskar DM^31^	2013	India	Not surgery	50	50	59.1 ± 13.4	59.5 ± 15.03	20 g MgSO4 over 24 h
Abbas SH^32^	2015	Pakistan	CABG	130	130	51.7 ± 10.2	51.7 ± 10.2	1 g MgSO4 over 24 h
Mohammadzadeh A^33^	2017	Iran	CABG	125	125	60.8 ± 7.6	61.3 ± 6.6	30 mg/kg MgSO4 in 5 min

Notes: Mg, magnesium; P, placebo; CABG, coronary artery bypass grafting

**Table 2.** Arrhythmias prevalence using Random Effect Meta-Analysis

Type of arrhythmia	Treatment	Number of studies	Prevalence%	Confidence interval 95% (CI%95)	Heterogeneity index I^2^ (%)	*P* value
Ventricular tachycardia	Magnesium	7	5.67	1.38–11.97	71.81	0.003
	placebo	7	15.04	6.47–26.06	88.9	0.00
Ventricular fibrillation	Magnesium	7	2.13	0.00–6.59	76.81	0.00
	Placebo	7	4.43	0.31–11.69	86.09	0.00
Total of Ventricular arrhythmia	Magnesium	13	11.88	6.71–18.17	82.99	0.00
	Placebo	13	24.24	14.52–35.43	92.11	0.00
Atrial fibrillation	Magnesium	9	9.72	3.31–18.63	92.15	0.00
	Placebo	9	22.37	15.86–29.59	85.3	0.00
Supraventricular tachycardia	Magnesium	6	4.90	0.84–11.28	75.36	0.00
	Placebo	6	14.62	7.26–23.82	78.76	0.00
	Magnesium	14	10.36	5.55–16.32	87.16	0.00
Total of supraventricular arrhythmia	Placebo	14	23.91	18.82–29.38	75.10	0.00
Bradycardia	Magnesium	4	6.46	0.71–12.21	78.9	0.00
	Placebo	4	7.2	1.03–1.37	79.8	0.00
Total Arrhythmia	Magnesium	22	41	11.44–21.0	85.89	0.00
	Placebo	22	30.85	25.07–39.63	86.21	0.00

### Ventricular arrhythmias (ventricular tachycardia or ventricular fibrillation)

Table 2 represents the total rate of arrhythmias after meta-analysis of the extracted data. As seen in this table, thirteen studies [12–14, 16–20, 23, 27, 29, 31, 32] included for evaluating the prevalence of the ventricular arrhythmia. The prevalence of ventricular tachycardia in the group receiving magnesium sulfate and placebo was 5.67% (95% CI, 1.38–11.97) and 15.04% (95% CI, 6.47–26.06) respectively. Moreover, the prevalence of ventricular fibrillation in the magnesium sulfate group was 2.13% (95% CI, 0.00–6.59) compared to 4.43% (95% CI, 0.31–11.69) in the placebo group. The total rate of ventricular arrhythmia was 11.88% (95% CI, 6.71–11.17) and 24.24% (95% CI, 14.52–35.43) within the magnesium and placebo groups respectively.

We also compared patients with and without arrhythmia in magnesium and placebo groups to determine the association between magnesium and the incidence of ventricular arrhythmias (Table 3).

**Table 3:** Magnesium administration and the incidence and type of arrhythmias compared to placebo

Type of fractures	Summary odds ratio (OR)	95% confidence interval	Between studies	
			I^2^	p for heterogeneity
Ventricular tachycardia Ventricular fibrillation Total of Ventricular arrhythmia	0.65 0.69 0.38	0.50–0.85 0.47–1.01 0.23–0.64	55.6% 48.9% 79.0%	0.035 0.098 0.000
Atrial Fibrillation Supraventricular tachycardia Total of Supraventricular arrhythmia	0.46 0.48 0.43	0.28–0.76 0.33–0.70 0.28–0.65	76.9% 48.5% 73.4%	0.000 0.084 0.000
Bradycardia	1.29	0.99–1.69	12.7	0.329
Total Arrhythmia	0.41	0.29–0.58	82.1	0.000

**Fig. 2:**
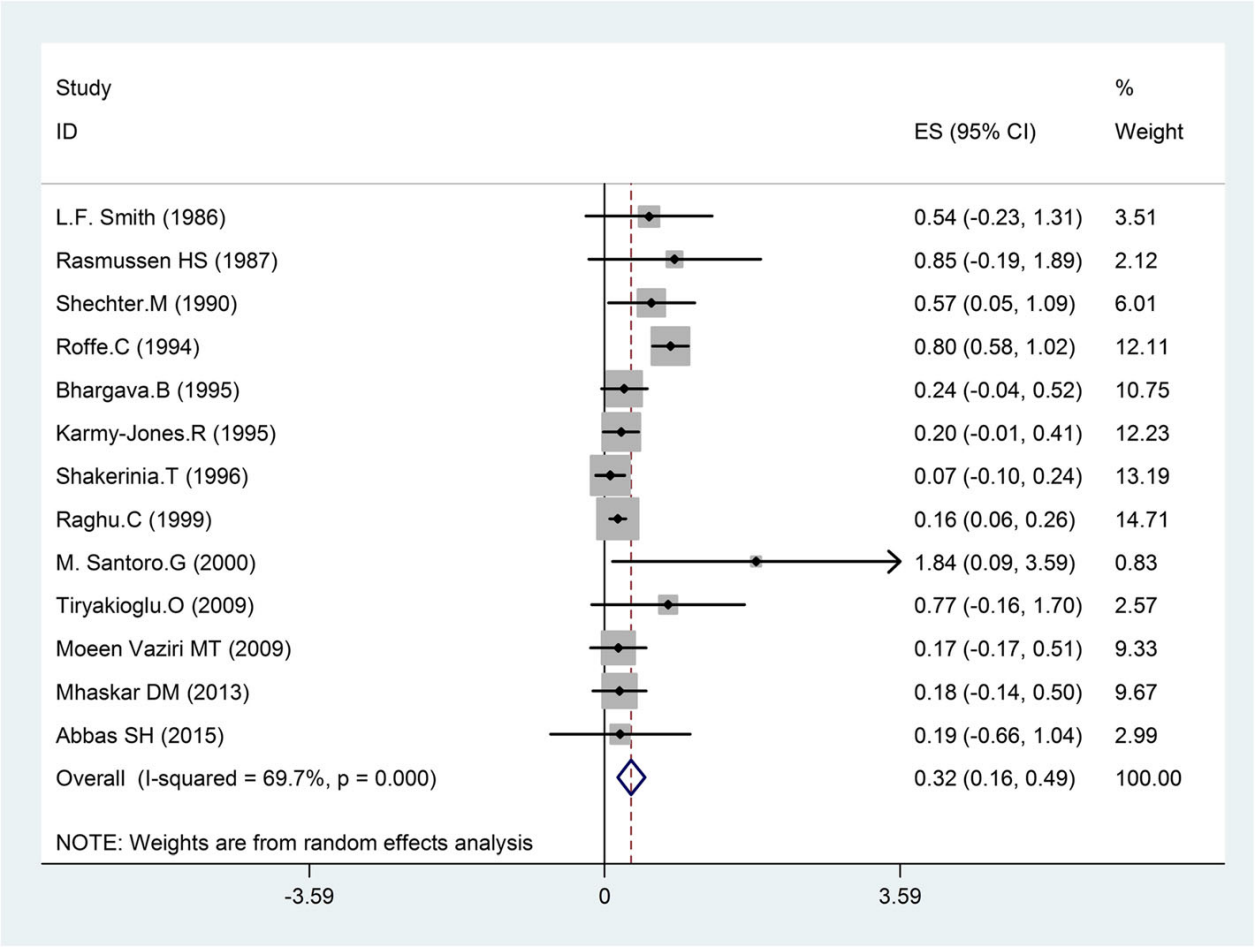
Forest plot and the rate of ventricular arrhythmias (left: magnesium, right: placebo).Square represents effect estimate of individual studies with their 95% confidence intervals with size of squares proportional to the weight assigned to the study in the meta-analysis. In this chart, studies are stored in order of the year of publication and author’s names, based on a random effects model

The present meta-analysis with a fixed-effect model showed no difference in ventricular fibrillation within the magnesium group compared with placebo (OR = 0.69, 95%CI, 0.47–1.01; I^2^ = 48.9%, *p* = 0.098); however a significant decrease observed in ventricular tachycardia between magnesium and placebo groups (OR = 0.65, 95% CI, 0.50–0.85; I^2^ = 55.6%, *p* = 0.035). By using random effects model this meta-analysis showed that magnesium could decrease ventricular arrhythmias compared with placebo (OR = 0.32, 95% CI 0.16–0.49; *p* < 0.001, Fig. 2). There was heterogeneity among trials (I^2^ = 69.7%; *p* = 0.000).

### Supraventricular arrhythmias (atrial fibrillation or supraventricular tachycardia)

As shown in Table 2, fourteen studies [13, 15–19, 22, 24, 26–28, 30–32] reported the prevalence of supraventricular arrhythmia. The incidence of arrhythmias was as follows: atrial fibrillation was 9.72% (95% CI, 3.31–18.63) within the magnesium sulfate group and 22.37% (95% CI, 15.86–29.59) within the placebo group. Supraventricular tachycardia was 4.90% in the magnesium group (95% CI, 0.84–11.28) and 14.62% in the placebo group(95% CI, 7.26–23.82). The total rate of supraventricular arrhythmia was 10.36% (95% CI, 5.55–16.32) and 23.91% (95% CI, 18.82–29.38) within the magnesium and placebo groups respectively.

**Fig. 3:**
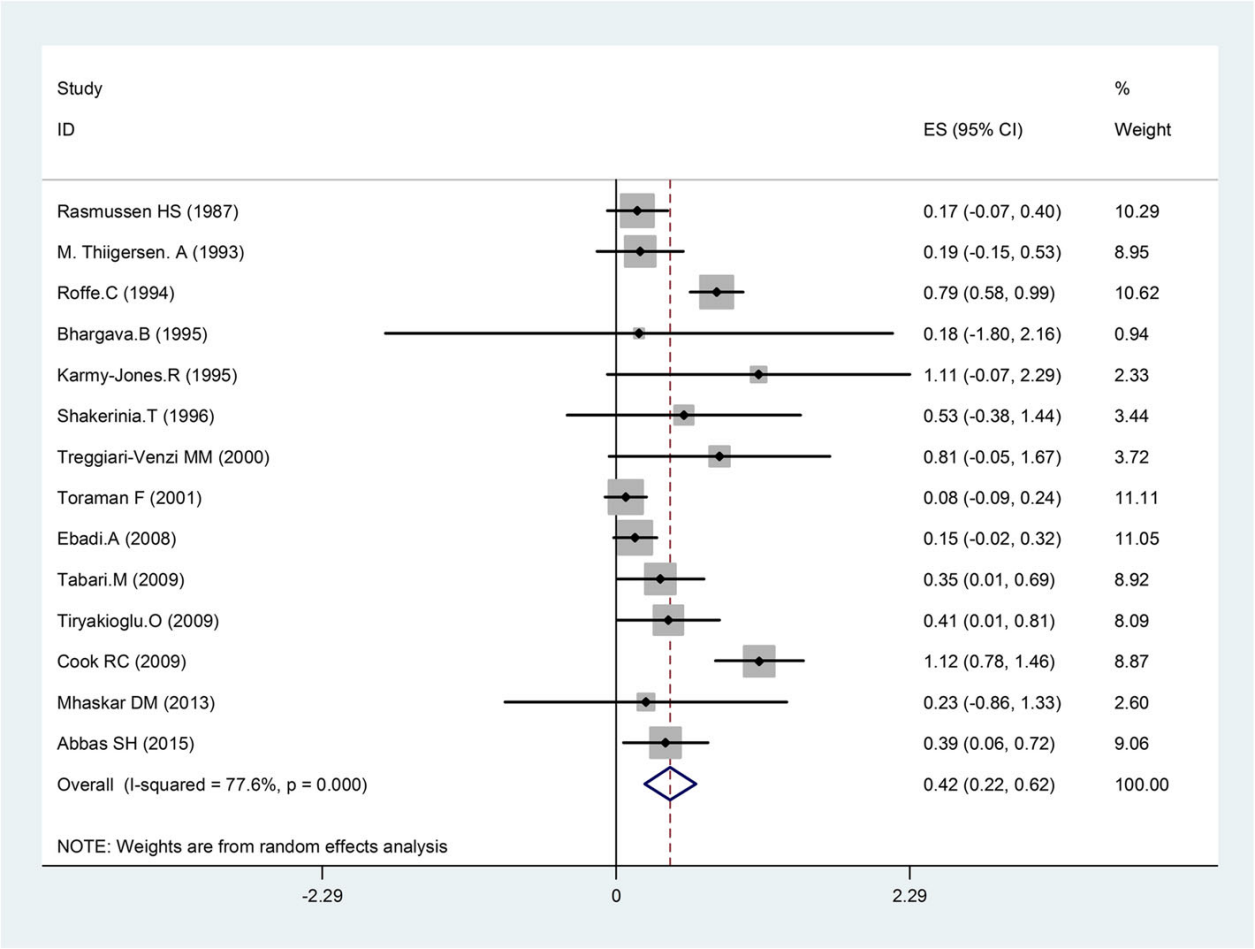
Forest plot and the rate of supraventricular arrhythmias (left: magnesium, right: placebo). Square represents effect estimate of individual studies with their 95% confidence intervals with size of squares proportional to the weight assigned to the study in the meta-analysis. In this chart, studies are stored in order of the year of publication and author’s names, based on a random effects model

Random effects analysis showed a significant decrease in atrial fibrillation comparing magnesium with placebo (OR = 0.46, 95%CI, 0.28–0.76; I^2^ = 76.9%, *p* = 0.000); also a reduction in supraventricular tachycardia observed via fixed-effect model (OR = 0.65, 95% CI, 0.50–0.85; I^2^ = 48.5%; *p* = 0.035). Overall, meta-analysis with random effects model showed that magnesium could decrease supraventricular arrhythmia compared with placebo (OR = 0.42, 95% CI 0.22–0.65; *p* < 0.001, Fig. 3). There was significant heterogeneity among trials (I^2^ = 77.6.4%; *p* = 0.000), (Table 3).

### Bradycardia

Among reviewed studies, four articles reported the prevalence of bradycardia. The rate of bradycardia in the magnesium sulfate and placebo groups was 6.46% (95% CI, 0.71–12.21) and 7.2% (95% CI, 1.03–1.37) respectively (Table 2). Random effects meta-analysis did not show a beneficial effect of magnesium on the reduction of bradycardia (OR = 1.29, 95% CI 0.99–1.69; *p* = 0.329). The reviewed studies showed limited evidence of heterogeneity (I^2^ = 12.7%; *p* > 0.001), (Table 3).

### Subgroup analyses

It was clear that some factors might influence the associations between magnesium and the incidence of cardiac arrhythmias. Therefore, we conducted subgroup analyses to minimize heterogeneity among various studies. In the current study, the effect of magnesium consumption versus placebo on the reduction of cardiac arrhythmias examined by the dosage of magnesium (< 10 g, > 10 g); fourteen trials used magnesium less than 10 g and eight used magnesium 10 g or more within the first 24 h. We found a significant decrease in cardiac arrhythmias comparing magnesium with placebo in both groups (magnesium< 10 g OR = 0.42, 95% CI: 0.24–0.59; I2 = 71.0%, *p* = 0.000 and magnesium ≥10 g OR = 0.34, 95% CI: 0.08–0.60; I2 = 90.1%, p = 0.000).

We also performed subgroup meta-analysis based on the clinical settings; surgery or not surgery. Nine trials evaluated the effect of magnesium consumption on the incidence of arrhythmias in nonsurgical patients. The results of the meta-analysis showed a significant decrease comparing magnesium with placebo (OR = 0.33, 95%CI, 0.10–0.57; I2 = 88.7%, *p* = 0.000). Also, thirteen trials performed in surgical patients and showed that magnesium consumption could have a positive effect in reducing cardiac arrhythmias (OR = 0.43, 95%CI 0.24–0.62; I2 = 73.3%, p = 0.000).

The results of subgroup analyses showed that the dose of magnesium used or the clinical settings did not affect the reduction of arrhythmias.

To find the source of heterogeneity a meta-regression performed. As seen in Table 4, the association between sample size, mean age, published year, the consumed dose of magnesium and the effect size of the outcomes evaluated. Our results showed that there was no significant association between the reduction of ventricular arrhythmias and sample size (*p* = 573), mean age (*p* = 553), published year (*p* = 283), and the consumed dose of the magnesium (*p* = 410).

**Table 4:** Source of heterogeneity by multivariate meta-regression analysis

Factors	Coefficient	Standard error	*P*
Published year	−.0121418	.010758	0.283
Sample size	−.0010426	.0017948	0.573
Mean age	.0118355	.0192186	*0.553*
Dose of Magnesium	.2159504	.0109332	*0.410*

### Publication Bias

**Fig. 4:**
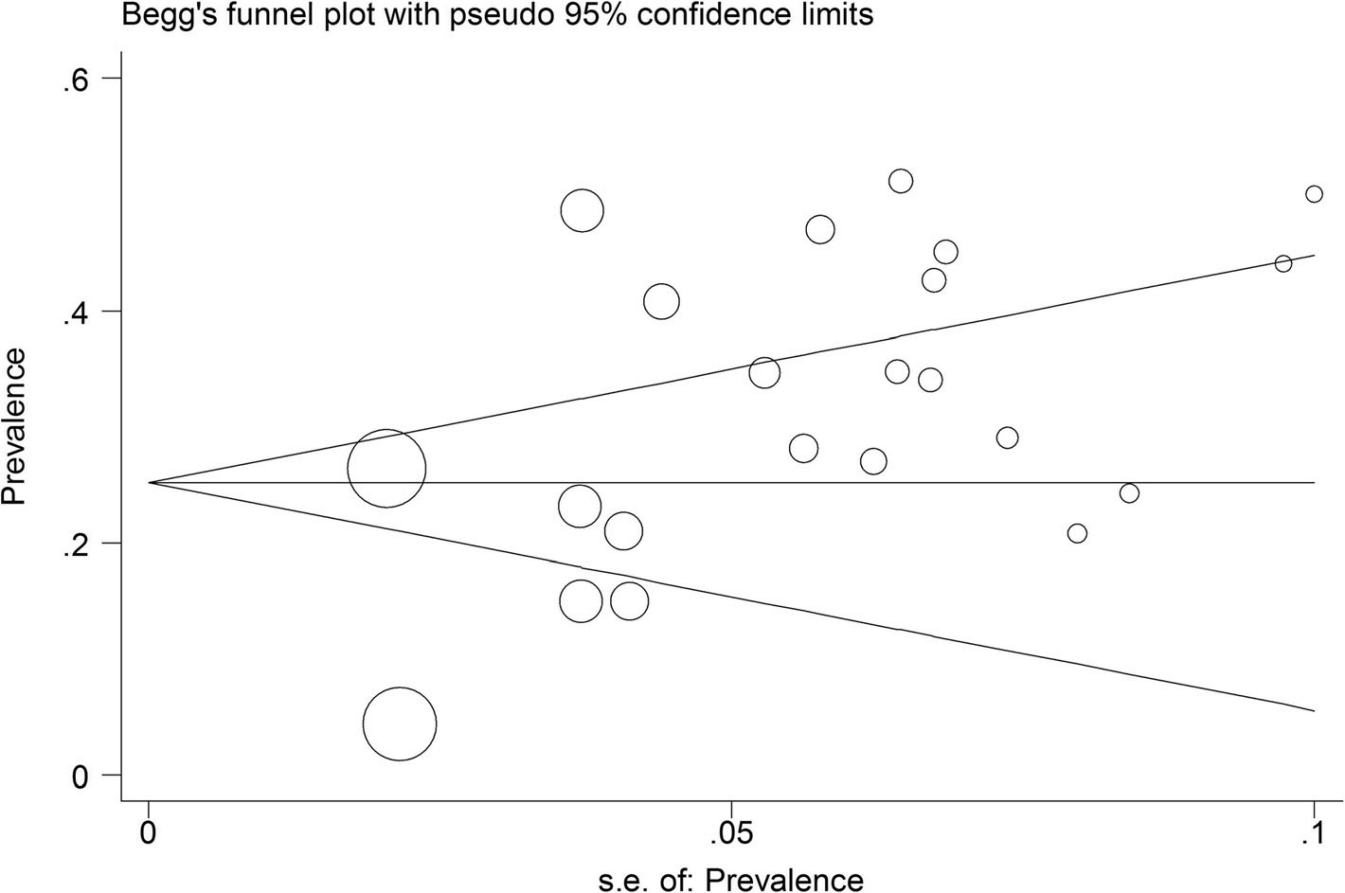
Begg’s funnel plot for publication bias in the risk difference analysis

Figure 4 presents the Begg’s funnel plot for publication bias in the risk difference analysis of the effect of magnesium in reducing ventricular arrhythmias. According to the publication bias figure, the effect of bias was not significant when Begg’s funnel plot evaluated (*p* = 0.204, Fig. 4); *P*-values for Egger’s regression asymmetry test were 0.008; thus, the Egger tests revealed the evidence of publication bias in this study.

## Discussion

Cardiac arrhythmia is caused by a wrong rate or rhythm of the heartbeat, which are under the control of the cardiac conduction system [34]. Magnesium sulfate is frequently used to reduce cardiac arrhythmias in patients with the acute coronary syndrome [35]. Numerous attempts are in action to diminish cardiac arrhythmias. This study is a meta-analysis of previously conducted studies comparing magnesium consumption versus placebo control in reducing the incidence of cardiac arrhythmia; the current systematic review and meta-analysis of these twenty-two randomize control trials found a positive correlation between the administrations of magnesium sulfate and the reduction of cardiac arrhythmias.

Our results were consistent with those of the previous meta-analyses which evaluated the effect of magnesium on the incidence of arrhythmias. A meta-analysis of eight trials included 930 patients with acute myocardial infarction, showed the beneficial effect of magnesium to prevent arrhythmias. Horner’s study showed that the administration of magnesium in acute myocardial infarction associated with 49% reduction in ventricular arrhythmias and 54% reduction in supraventricular tachycardia [36]. A meta-analysis of seventeen trials with 2069 patients reported that magnesium administration could reduce the risk of supraventricular arrhythmias by 23% (atrial fibrillation by 29%) and of ventricular arrhythmias by 48% after cardiac surgery [37]. Our meta-analysis showed that magnesium could decrease the risk of ventricular arrhythmias about 32% and supraventricular arrhythmias about 42% respectively.

One of our results was that magnesium sulfate could reduce the incidence of supraventricular arrhythmias more than ventricular arrhythmias. However, the effect of magnesium in reducing the incidence of ventricular arrhythmias has not investigated as widely as the same for supraventricular arrhythmias. A meta-analysis of twenty studies with 3696 patients who underwent coronary artery bypass did not find any effect of magnesium on the incidence of ventricular arrhythmias; the authors identified the effect of magnesium sulfate in reducing postoperative supraventricular arrhythmias when examined by lower-quality studies [38].

This study suggests that magnesium sulfate administration reduces supraventricular arrhythmia. Moreover, the observed effects were greater for atrial fibrillation. The most common arrhythmia after coronary artery bypass graft is atrial fibrillation. Alghamdi and colleagues in a meta-analysis of eight randomized controlled trials revealed that the use of intravenous magnesium associated with a significant reduction in the incidence of atrial fibrillation after coronary artery bypass surgery [39]. Another meta-analysis of twenty-two trials with 2896 patients showed that there was an overall reduction in atrial fibrillation after magnesium administration [40]. A meta-analysis of 2490 patients from twenty randomized trials concluded that magnesium administration could be an effective prophylactic measure for prevention of the postoperative atrial fibrillation [41]. Another meta-analysis of seven clinical trials with 1028 participants revealed that intravenous magnesium reduced the incidence of postoperative atrial fibrillation about 36% [42]. Many of the conducted studies encouraged the use of intravenous magnesium to prevent postoperative atrial fibrillation after coronary artery bypass grafting. The present meta-analysis was consistent with previous studies; we confirmed the previous results and concluded that magnesium administrating could be useful in prevention and treatment of various cardiac arrhythmias.

As discussed above, several meta-analyses have explored the effects of magnesium administration and cardiac arrhythmias, but there was also data not included. Furthermore, there have been further developments and newer trials since the publication of previous meta-analyses, which did not include these trials. In fact, this study is also the largest one which examined the effect of magnesium on the incidence of cardiac arrhythmias.

Magnesium plays an essential role in many fundamental biological processes, for example, it participates in many enzymatic reactions and many ion channels functions [34]. Magnesium also is a cofactor of the membrane Na-K pump; it regulates the outward K+ movement and potassium is transported equally in both directions when Mg2+ is absent [43, 44]. Magnesium deficiency can reduce the amount of intracellular K+ and the pump’s activity, which leads to partial depolarization and changes in the activity of many potential-dependent membrane channels [43, 44]. So, its deficiency also disturbs the resting membrane potential of the cardiac cells and results in cardiac arrhythmias [44].

Hypomagnesaemia is common among patients with cardiovascular diseases [45]. Therefore, measurement of the serum magnesium level of patients with cardiovascular disease before surgery is necessary and indicate injection of magnesium sulfate to prevent these complications. Hypomagnesaemia in cardiovascular disease may occur due to various reasons such as treatment of hypertension with diuretics, diabetic patients, and patients with cardiomyopathy [46].

Magnesium deficiency is an important factor responsible for supraventricular and ventricular arrhythmias [34]. Beluri and colleagues found that the risk of ventricular arrhythmias in magnesium deficiency increased dramatically and suggested that hypomagnesemia could be considered one of the most important causes of ventricular arrhythmias [47]. Hypomagnesemia in patients with congestive heart failure causes arrhythmia. Fall Solomon and colleagues found that 55% of patients with congestive heart failure suffer hypomagnesemia, which results in ventricular premature beats and atrial fibrillation [48].

All these findings indicate the importance of serum magnesium monitoring level in cardiac patients. Therefore, by checking and correcting serum magnesium level, it is possible to prevent many cardiac arrhythmias and improve the care of cardiac patients.

This meta-analysis had several limitations. First, there was a lack of uniformity in the reviewed trials regarding the clinical settings and the amount of magnesium administered. For example, some study was among revascularized patients (CABG or angioplasty) while others in non-surgical patients; however, we performed a meta-analysis and concluded that the effect of magnesium on the incidence of arrhythmias not affected by either clinical settings or the amount of magnesium administered. Secondly, we were unable to conduct a subgroup analysis regarding the concurrent use of other antiarrhythmic medications. Insufficient available data about the concurrent use of other antiarrhythmic agents, which could modify effect size, prevented us from the evaluation of such cases. Third, in a meta-analysis sample size and standard deviation are very important in combining the results of studies and may be influenced the evaluated outcomes. Fourth, the possible effects of publication bias inherent in any meta-analysis could not be ruled out. Finally, some identified studies presented defective quantitative data and could not be included in this meta-analysis.

## Conclusion

The present meta-analysis showed that the total rate of cardiac arrhythmia was significantly lower in the group receiving magnesium sulfate than placebo The current finding also showed that magnesium consumption would decrease ventricular and supraventricular arrhythmias compared with placebo. In conclusion, our study suggested that administration of magnesium sulfate could be safe, effective and cost-effective in the prevention of many cardiac arrhythmias. Therefore, by checking and correcting serum magnesium level, it may be possible to prevent a large proportion of cardiac arrhythmias and improve cardiac patient’s health. However, other studies should be done about the dose and the time of adding magnesium sulfate until proved that this method is effective in the prevention of cardiac arrhythmias.

## Abbreviations

CABG: Coronary Artery Bypass Grafting
PCI: Percutaneous Coronary Intervention
